# Detection of volatile organic compounds in headspace of *Klebsiella pneumoniae* and *Klebsiella oxytoca* colonies

**DOI:** 10.3389/fped.2023.1151000

**Published:** 2023-11-24

**Authors:** Michelle Bous, Malte Tielsch, Cihan Papan, Elisabeth Kaiser, Regine Weber, Jörg Ingo Baumbach, Sören L. Becker, Michael Zemlin, Sybelle Goedicke-Fritz

**Affiliations:** ^1^ Department of General Pediatrics and Neonatology, Saarland University, Homburg, Germany; ^2^ Centre for Infectious Diseases, Institute of Medical Microbiology and Hygiene, Saarland University, Homburg, Germany; ^3^ Department Bio- and Chemical Engineering, Technical University Dortmund, Dortmund, Germany

**Keywords:** volatile organic compounds, ion mobility spectrometry, premature infant, biomarkers, *klebsiella pneumoniae*, detection, pediatrics, non-invasive diagnostics

## Abstract

**Introduction:**

Early diagnosis of infections and sepsis is essential as adequate therapy improves the outcome. Unfortunately, current diagnostics are invasive and time-consuming, making diagnosis difficult, especially in neonatology. Novel non-invasive analytical methods might be suitable to detect an infection at an early stage and might even allow identification of the pathogen. Our aim is to identify specific profiles of volatile organic compounds (VOCs) of bacterial species.

**Methods:**

Using multicapillary column-coupled ion mobility spectrometry (MCC/IMS), we performed headspace measurements of bacterial cultures from skin and anal swabs of premature infants obtained during weekly screening for bacterial colonization according to KRINKO. We analyzed 25 *Klebsiella pneumoniae* (KP) cultures on MacConkey (MC) agar plates, 25 *Klebsiella oxytoca* (KO) cultures on MC agar and 25 bare MC agar plates as a control group.

**Results:**

Using MCC/IMS, we identified a total of 159 VOC peaks. 85 peaks allowed discriminating KP and bare MC agar plates, and 51 peaks comparing KO and bare MC agar plates and 6 peaks between KP and KO *(*significance level of *p* < 0.05 after *Bonferroni post hoc analysis)*, respectively. Peaks P51 (*n-Decane*) and P158 (Phenylethyl Alcohol), showed the best sensitivity/specificity/ positive predictive value/negative predictive value of 99.9% each (*p* < 0.001) for KP. P158 showed the best sensitivity/specificity/positive predictive value/negative predictive value of 99.9% each (*p* < 0.001) for KO*.* Comparing KP and KO, best differentiation was enabled using peaks P72, P97 and P16 with sensitivity/specificity/positive predictive value/negative predictive value of 76.0%, 84.0%, 82.6%, 77.8%, respectively (*p* < 0.05).

**Discussion:**

We developed a method for the analysis of VOC profiles of bacteria. Using MCC/IMS, we demonstrated that VOCs derived from bacteria are clearly distinguishable from a bare agar plate. Characteristic peaks obtained by MCC/IMS are particularly suitable for the species-specific identification and differentiation of KP and KO. Thus, MCC/IMS might be a useful tool for *in vitro* diagnostics. Future studies must clarify whether similar patterns of VOCs can be detected *in vivo* in patients that are colonized or infected with KP or KO to enable rapid and accurate diagnosis of bacterial colonization.

## Introduction

Prematurity includes a state of immaturity of all organ systems including the immune system. Thus, preterm infants are at high risk for nosocomial infections. Therefore, weekly colonization screening is recommended by German Commission on Hospital Hygiene and Infection Protection (KRINKO) at the Robert Koch Institute (RKI) since 2013 as bloodstream infections and bacterial colonization pose a significant risk of morbidity and mortality for preterm infants (1). As infections of preterm infants often manifest with nonspecific symptoms, diagnosis is difficult and the time window to achieve adequate diagnosis and treatment is short. The existing diagnostic tests require painful procedures and are often linked to high costs and to limited sensitivity. Presently, acute inflammatory markers provide limited sensitivity, especially at the onset of an infection. Time consuming laboratory tests can delay adequate treatment for hours triggering empiric antibiotic treatment before knowing the laboratory results. A fast and real-time diagnostic tool could reduce unnecessary exposure to antibiotics in suspected, but unconfirmed sepsis. Rapid diagnosis and prompt initiation of therapy significantly improves outcome (2–4).

The analysis of volatile organic compounds (VOCs) represents an innovative approach for non-invasive diagnostics (5). VOCs can be produced by the host or by microbes and are emitted via body secretions or breath. Alterations in specific VOC profiles are linked to perinatal or neonatal diseases (6). Previous studies revealed the high potential of VOC analysis and its application for non-invasive diagnostics (6–9). We have developed a method using ion mobility spectrometer coupled to multi capillary columns (MCC/IMS) to measure VOC profiles and to assign individual VOCs to biochemical markers using a reference data set (BS-MCC/IMS-analyses database). Using MCC/IMS, characteristic VOC profiles can be detected in the incubator atmosphere of neonates (10). Moreover, using VOC analysis via MCC/IMS, we found that 5-methyl pentane as a potential biomarker for chorioamnionitis, a common cause of prematurity (11).

Bacteria can also produce VOCs; some VOCs originate exclusively from certain bacterial species, and analysis of VOCs profiles enables distinguishing between several bacterial species (9, 12, 13). Also, there are changes in VOCs profiles of neonates suffering from late-onset sepsis (14). Common healthcare-associated pathogens are Gram-negative bacteria such as *Klebsiella pneumoniae* (KP) and *Klebsiella oxytoca* (KO). The detection of KP is of the utmost importance as it can cause neonatal infections and sepsis, which may be difficult to treat due to the prevalence of multi-drug resistant strains (15–19). Similarly, KO can acquire antibiotic resistance and cause outbreaks in neonatal intensive care units (NICU) (20–22). The occurrence of KO is linked to antibiotic associated hemorrhagic colitis (23) and might be associated with necrotizing enterocolitis (24).

The aim of this study was to establish headspace measurements of KP and KO independent from ambient air for measuring and distinguishing bacterial cultures originating from routine anal/rectal swabs of preterm infants using MCC/IMS as a novel noninvasive, rapid and precise method.

## Methods

### Patients

This study was performed at the Department of Pediatrics, Saarland University Medical Center, Homburg (Germany) and at the Institute of Medical Microbiology and Hygiene, Saarland University, Homburg (Germany) after approval by the Ethical committee Saarland (reference 276/17) from April 2021 to October 2021. All acquired data were recorded and processed in an anonymized form.

### Sample collection and processing

Microbial cultures of KP and KO on MacConkey (MC) agar plates (Becton, Dickinson and Company, Franklin Lakes, New Jersey, USA) originated from rectal/anal swabs of preterm infants of the NICU of the Department of Pediatrics, Saarland University Medical Center, Homburg (Germany). The swabs were taken twice a week as part of the routine screening on bacterial colonization. Swabs were transferred in eSwab™ transport medium (Copan, Brescia, Italy). In the Institute of Medical Microbiology and Hygiene, Saarland University, Homburg (Germany), swabs were spread out onto MC agar plates. Only those agar plates with growth of KP and KO cultures were included in this study. Following a Standard operating procedure (SOP), samples were processed as follows: a part of the agar with bacterial colonies on it (size: 0.5 × 0.5 cm) was cut out and placed into a laboratory bottle.

### Data assignment/headspace measurement

**Figure 1:**
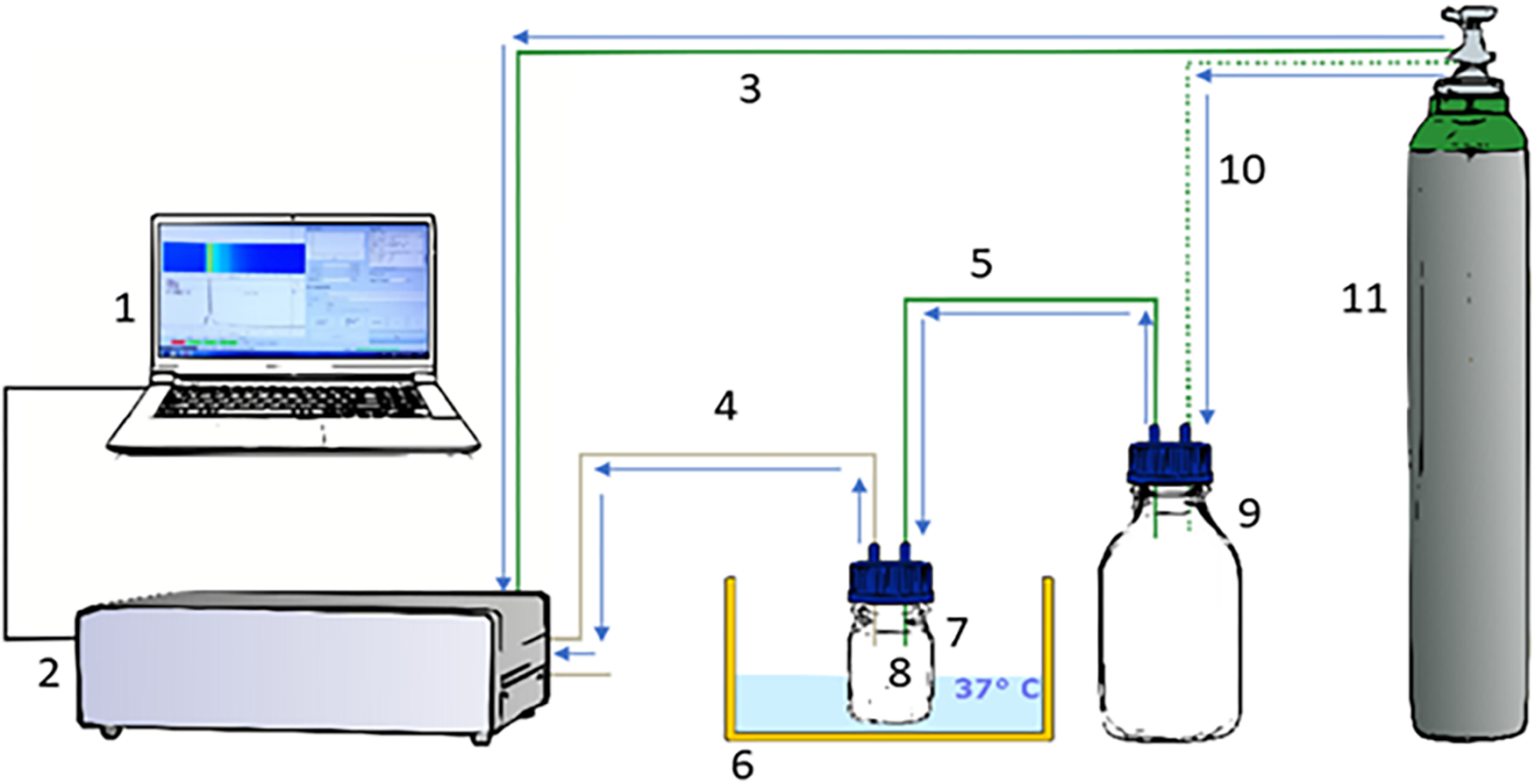
Experimental design. We used an MCC/IMS BreathDiscovery (B & S Analytik GmbH, Dortmund, Germany) to perform headspace measurements of VOCs derived from the fecal und urine samples. (1) Laptop; (2) MCC/IMS BreathDiscovery (B & S Analytik GmbH, Dortmund, Germany); (3) Connecting tube between MCC/IMS and synthetic air supply gas bottle; (4) Connecting tube between MCC/IMS and sample container; (5) Connecting tube between gas reservoir and sample container; (6) Water bath; (7) Sample container: small laboratory bottle (100 ml); (8) Diaper sample; (9) Gas reservoir: large laboratory bottle (1,000 ml); (10) Connecting tube between synthetic air supply gas bottle and gas reservoir; (11) Synthetic air supply gas bottle; PFA tubes (green) as drift gas supply; PFA tube (grey) as sample input. Data acquisition was directed and recorded using VOCan v3.7 (B & S Analytik GmbH, Dortmund, Germany).

To perform headspace measurements of VOCs derived from bacterial colonies, we used an MCC/IMS BreathDiscovery (B & S Analytik GmbH, Dortmund, Germany). It was placed on a metal cart with a laptop computer and connected to a synthetic air supply. The pre-separation was performed using an OV-5 (5% - diphenyl, 95% - dimethylpolysiloxane) multi-capillary column (MCC) (Multichrom Ltd., Novosibirsk, Russia). The device and sampling parameters are given in Supplementary Table 1. The methods for VOC analysis were published earlier. A laboratory bottle (100 ml) heated to 37°C served as a sample container. A closed system was established: A large laboratory bottle (1,000 ml, gas reservoir, Schott Duran®, DURAN Group GmbH, Wertheim /Main, Germany) was connected to the small bottle (100 ml, Schott Duran®, DURAN Group GmbH, Wertheim /Main, Germany) via a perfluoroalkoxyalkane (PFA) tube that was led through the caps. Both bottles were filled with synthetic air as carrier gas. Another tube connected the cap of the small laboratory bottle and the sampling input of MCC/IMS device (Figure 1). Data was acquired using VOCan v3.7 (B & S Analytik GmbH, Dortmund, Germany). The standard operation conditions are used as recommended by the supplier of the instrument. IMS was regularly calibrated using standardized reference mixture (“R06” calibration liquid, B & S Analytik GmbH, Dortmund, Germany, Supplementary Table 2).

### Statistical analysis

We evaluated the data acquired by MCC/IMS using the software VisualNow 3.7 (B & S Analytik GmbH, Dortmund, Germany). All peaks were characterized by their specific combination of retention time and drift time (corresponding 1 / K_0_-value, see Supplementary Table 3). The databank layer (BS-MCC/IMS-analyses database) was used for peak referencing and determination of retention times and 1/K_0_-values. Here, pure analytes were measured 10 times each and comparison with parallel measurements using GC/MS standard procedures was performed (25, 26). Peak intensity (in volts) was considered as an indirect measure of compound concentration. A specific threshold was calculated for each peak and comparison. Box-and-Whisker plots and a rank sum test using Mann-Whitney-U test and Bonferroni *post hoc* analysis correction were performed. The *n*-value was set at *n* = 25 for KP, *n* = 25 for KO and *n* = 25 for bare MC agar plates. The *α*-level was defined to be 0.05, the *p*-value (one-tailed) was determined to be <0.05. Significant peaks [*p* < 0.05, 95% confidential interval (CI)] were used for further evaluation with decision trees (DT).

## Results

**Figure 2:**
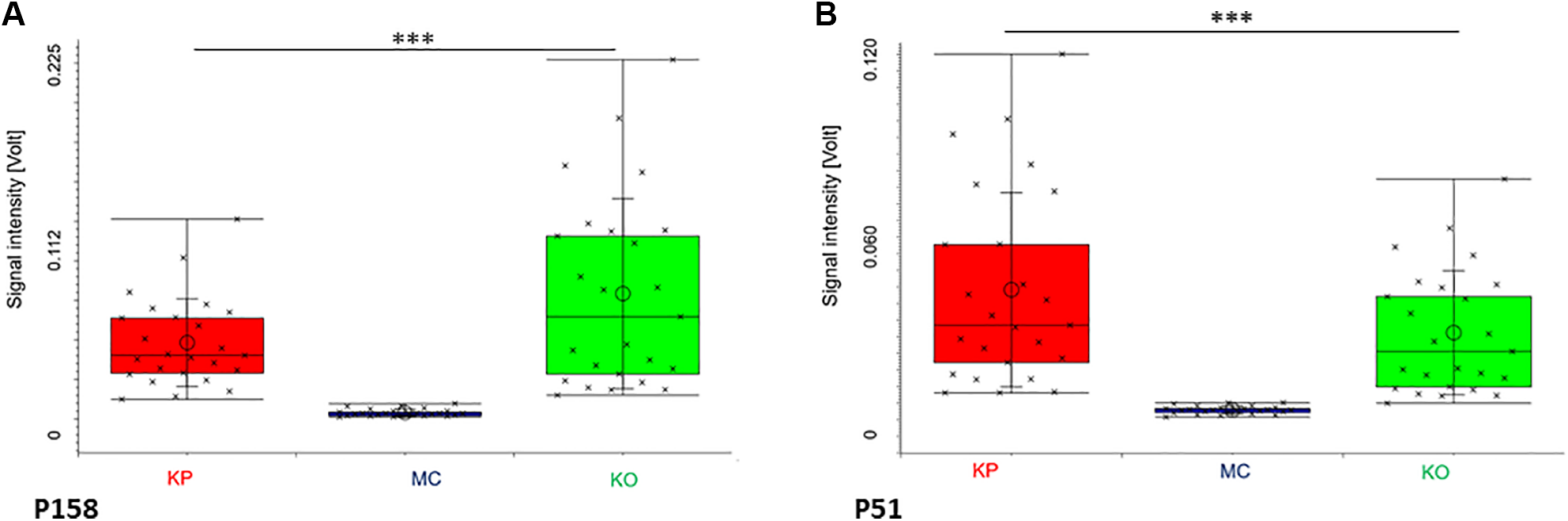
Box-and-whisker-Plot of the peaks P158 (**A**) and P51 (**B**). After Bonferroni *post hoc* analysis correction, Peaks P51 and P158 showed the highest sensitivity, specificity, positive and negative predictive value with 99.9% each in a significance level with *p* < 0.001 for *K. pneumoniae* (KP) when comparing KP and bare MacConkey agar plates (MC). Comparing *K. oxytoca* (KO) and MC P158 reaching the highest sensitivity, specificity, positive and negative predictive value of 99.9% each in a significance level with *p* < 0.001.

We analyzed a total of 75 agar plates with KP (*n* = 25) or KO (*n* = 25) and bare MC agar plates (*n* = 25), respectively. We identified 159 signals (peaks) from each comparison such as 85 peaks between bare MC agar plates and KP, 51 peaks between bare MC agar plates and KO and 6 between KP and KO, respectively. We found 148 peaks exclusively assigned to KO or KP and 11 peaks exclusively assigned to bare MC agar plates. 17 peaks occurred in all three groups (MC/KO/KP) and did not differ significantly between the individual groups. When comparing KP and bare MC agar plates, peak P51 and P158 showed the highest sensitivity, specificity, positive and negative predictive value after Bonferroni *post hoc* analysis correction (sensitivity and specificity 99.9%, respectively, *p* < 0.001) for KP. Comparing KO and bare MC agar plates peak P158 reaching the highest sensitivity, specificity, positive and negative predictive value of 99.9% each in a significance level with *p* < 0.001 (Figure 2). Regarding the comparison between KO and KP*,* best differentiation was enabled using peaks P72, P97 and P16 with sensitivity, specificity and positive and negative predictive value of 76.0%, 84.0%, 82.6%, 77.8%, respectively in a significance level with *p* < 0.05 after Bonferroni *post hoc* analysis correction (Table 1).

**Table 1:** Statistical analyses for the model set and validation set for peaks P158 and P51.

	P158			P51		
Best direction	**KP > MC**	**KO > MC**	**KO > KP**	**KP > MC**	**KP > KO**	**KO > MC**
Best threshold	0.012	0.015	0.082	0.008	0.017	0.007
Classified right	50	50	35	50	31	49
Classified wrong	0	0	15	0	19	1
True positive	25	25	23	25	19	25
False positive	0	0	13	0	13	1
True negative	25	25	12	25	12	24
False negative	0	0	2	0	6	0
Sensitivity [%]	99.9	99.9	92	99.9	76	99.9
Specificity [%]	99.9	99.9	48	99.9	48	96
Positive predictive value [%]	99.9	99.9	63	99.9	59	96.2
Negative predictive value [%]	99.9	99.9	86	99.9	67	99.9
Accuracy [%]	100	70	70	100	62	98
Significance level (Mann Whitney *U*)	<0.001	<0.001	/	<0.001		<0.001
Significance level (Bonferroni correction)	<0.001	<0.001	/	<0.001		<0.001

**Figure 3:**
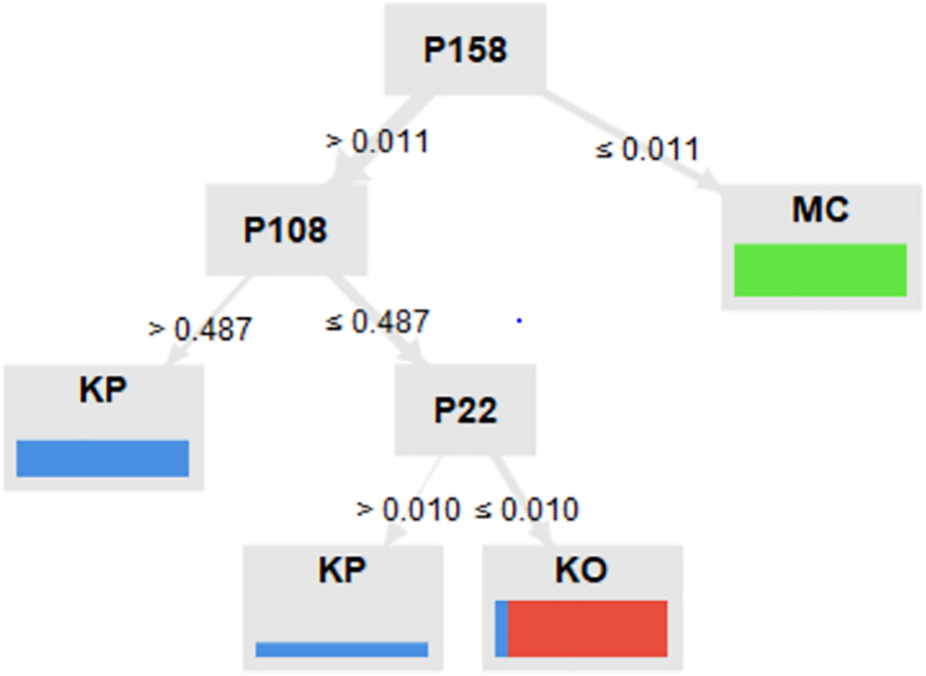
Decision tree. A total of three peaks enabled the differentiation of *K. pneumoniae* (KP), *K. oxytoca* (KO) and bare MacConkey agar plates (MC) regarding their signal intensities. With a signal intensity ≤0.011 V, P158 allows to differentiate between bare MC, KP and KO, respectively. At a signal intensitiy >0.487 V, KP is identified via P108. Peak P22 enables further differentiation regarding a signal intensity ≤0.487 V: a signal intensity ≤0.010 V represents KO and a signal intensity >0.005 V represents KP.

We established a decision tree using three peaks that enabled the differentiation of KP, KO and bare MC agar plates regarding their signal intensities. The peak P158 allows to differentiate between bare MC agar plates (with a signal intensity ≤0.011 V), KP and KO (with a signal intensity >0.011 V), respectively. Regarding signal intensities >0.487 V, KP is identified via P108. The Peak P22 enables further differentiation regarding a signal intensity ≤0.487 V: a signal intensity ≤0.010 V stands for KO and a signal intensity >0.005 V for KP (Figure 3).

Using our standardized database (B. Braun Melsungen-Database/BS-MCC/IMS-analyses database), two peaks were assigned to biochemical substances: P51 was assigned to *n-Decane* (CAS number 124-18-5) and P158 to Phenylethyl Alcohol (CAS number 60-12-8).

## Discussion

Premature babies show an increased susceptibility to nosocomial infections. Gold standard to verify bacterial bloodstream infections are blood cultures which yield results 24–48 h or even later (27–29). Rapid diagnosis and prompt initiation of therapy significantly improves outcome (2–4). In the present study we showed that KP and KO can be distinguished based on their VOC profile using MCC/IMS. As electronic nose devices detect patterns of VOC profiles, we chose MCC/IMS to precisely identify substances. In our study, we identified two signals that could be assigned to substances using a database (B. Braun Melsungen-Database/ GC/MS-MCC/IMS-analyses database): *n-Decane* and Phenylethyl Alcohol. Phenylethyl Alcohol is considered a metabolite in the Ehrlich Pathway and might derive from *Klebsiella* spp. Our future aim is to enable early diagnosis of neonatal infections by means of alterations in the VOC profile of neonates suffering from blood stream infections to be able to initiate quickly a targeted antibiotic therapy. Therefore, in the first step, we created the bacteria-specific VOC profiles using MCC-IMS in agar plates deriving from routine swabs. The next step will be the analysis of skin swabs themselves without any cultivation to differentiate KO/KP swabs from commensal bacteria – later we plan to perform measurement directly inside the incubator. As bacterial colonization is a risk factor for invasive infections, it would be favorable to quickly know about the respective colonization status without performing potential stressful swabs. If this is successful, we can focus on detecting infections in future studies.

VOC detection studies showed the great potential of VOC analysis with a wide array of techniques like breath analysis, headspace measurement of bio samples and the measurement of incubators atmosphere (7, 8, 10, 30). VOC analysis can be performed by several methods, e.g., gas chromatography coupled with mass spectrometry (GC-MS), gas chromatography time of flight- mass spectrometry (GC-ToF-MS), selected ion flow tube- mass spectrometry (SIFT-MS) and ion molecule reaction mass spectrometry (IMR-MS). Obtaining time series every 15 min is an advantage of MCC/IMS, especially in comparison to different mass spectrometric methods. The moisture content (e.g., exhaled breath samples) is a major limitation for most analytical methods, but not for MCC/IMS. On the other hand, bed side and on-site applications of MCC/IMS were reported since 2017 as medical products based on MCC/IMS technology were introduced into the market. Electronic nose devices using sensor technologies are based on pattern recognition and measurements can be conducted easily and at the bedside. As those devices do not allow qualitative VOC analysis, we decided to use an ion mobility spectrometer coupled to multi capillary columns (MCC/IMS) allowing the measurement of VOC profiles and the specification of individual VOCs using a reference data set. MCC/IMS analyses provide results within a few minutes and could therefore be used for instant bedside diagnostics. Electronic noses have been used to distinguish several bacterial strains from each other *in vitro* (9). *P. aeruginosa* was detected in human sputum and in culture via gas chromatography-mass spectrometry (GC-MS) (31). Using MCC/IMS Junger et al. showed it was also possible to distinguish individual bacterial strains based on their VOCs in pure cultures (13). We conclude that treatment and diagnostics on NICU are often linked to invasive and time-consuming procedures. Establishing a noninvasive, painless method by VOC analysis would be best for vulnerable patients like preterm infants. We were the first study to use routine swabs from preterm infants and to analyze grown pathogens on agar plates. We analyzed grown agar plates, but also empty agar plates for adjustment. Incubation of inoculated agar plates took place in microbiological incubators. In preliminary work, we also incubated empty agar plates to rule out influences of the incubator storage on the VOC profile. Further strengths of our study are that our measurements can be conducted quickly within a few minutes, even bedside diagnostics are feasible (10). MCC/IMS allows to detect substances with extremely low concentrations (pg/L) (5, 32) which enables detecting weak alterations in the VOC profile. However, due to the high sensitivity of the MCC/IMS, there is a high risk that ethanol and other environmental VOCs will interfere with the detection of VOCs that would be important for detection purposes, including bacteria. Our method uses a closed system to avoid influences by ambient air and other environmental influences. For this method, especially when developed further so it can be run without the need for an overnight culture, we would not need further consumables besides the costs for the synthetic air. Thus, this approach has the potential to be cost-effective and ecologically sustainable. Nevertheless, the present study was performed under laboratory conditions which may differ from the clinical setting. An inconvenience of our study is the measurement of bacterial colonies (KP/KO) on agar plates. The cultivation of the bacteria still takes time. For this reason, additional studies are planned to allow direct analyses of standard swabs taken from preterm infants. At this point, it is unclear if we can distinguish bacterial colonization from invasive infections via VOC analysis, these conditions may differ regarding their VOC profile based on different inflammatory responses. As bacterial colonies originate from swabs, there is an uncertainty about pure cultures of bacteria. A higher number of swab samples and measurements must be considered. *Klebsiella* spp. are a common cause of neonatal sepsis and they frequently carry resistance genes that render them multidrug-resistant (33). Currently, antibiotic susceptibility testing using electronic nose devices is still unproven. Hence, bacterial culture linked to antibiotic susceptibility testing currently remains gold standard. Under clinical conditions, influences like oral hygiene, food habits/nutrition, age and gender were found to influence VOC profile. Nevertheless, environmental influences could be reduced following standard operating procedures.

We showed that KP and KO can be distinguished based on their VOC profile using MCC/IMS. We achieved a first step to provide a supportive tool that might quickly detect bacterial colonization on NICU.

## Data Availability

The raw data supporting the conclusions of this article will be made available by the authors, without undue reservation.
