# Supplementary material for: Detection of volatile organic compounds in headspace of Klebsiella pneumoniae and Klebsiella oxytoca colonies
Source: Front Pediatr. 2023 Nov 24;11:1151000. doi: 10.3389/fped.2023.1151000 (PMC10704455; doi:10.3389/fped.2023.1151000)
Supplement: Supplementary file 1 [file Datasheet1.docx]

Supplementary Material

**Detection of volatile organic compounds in headspace of *Klebsiella pneumoniae* and *Klebsiella oxytoca* colonies**

**Michelle Bous, Malte Tielsch, Cihan Papan, Elisabeth Kaiser, Regine Weber, Jörg Ingo Baumbach, Sören L. Becker, Michael Zemlin, Sybelle Goedicke-Fritz**

*** Correspondence:**

Corresponding Author:

Sybelle Goedicke-Fritz, PhD

Saarland University Children's Hospital

Medical Faculty, University of Saarland

Phone: +49 6421-58 62650

E-Mail: Sybelle.goedicke-fritz@uks.eu

**Supplementary TABLE 1:** Device and sampling parameters

| **Carrier Gas Ionisation** | 63Ni (95 MBq) |
| --- | --- |
| **Column Ion Separation Drift tube** | 330 V/cm, 0.12 m |
| **Column temperature** | 40 °C |
| **Detection mode** | Positive |
| **Drift flow** | 99,9 mL/min |
| **MCC Flow** | 150 mL/min |
| **Multi Capillary Column (MCC)** | OV-5 |
| **Sample Flow** | 99,9 mL/min |
| **Sampling duration** | 20 s |
| **Synthetic air** | 20.5 ± 0.5% O2 in N2, purity ≥ 99,999 mol%) |

**Supplemental TABLE 2: Composition of R06 calibration liquid.** The R06 calibration liquid consists of six components. For each analyte, the chemical abstracts service (CAS) registry number is indicated in the table**.**

**Supplement TABLE 3:** Peaks were characterized by their specific combination of position retention time (RT) per second and drift time (corresponding 1/K0-value)

| **Name** | **1/K0** | **RT** | **1/K0 radius** | **RT radius** |
| --- | --- | --- | --- | --- |
| P0 | 0,761 | 105,5 | 0,003 | 7,2 |
| P1 | 0,761 | 134,5 | 0,003 | 8,4 |
| P2 | 0,775 | 56,5 | 0,003 | 5,3 |
| P3 | 0,758 | 57,0 | 0,003 | 5,3 |
| P4 | 0,728 | 56,5 | 0,003 | 5,3 |
| P5 | 0,686 | 17,0 | 0,003 | 3,7 |
| P6 | 0,652 | 9,0 | 0,003 | 3,4 |
| P7 | 0,628 | 13,5 | 0,003 | 3,5 |
| P8 | 0,635 | 21,5 | 0,003 | 3,9 |
| P9 | 0,620 | 25,0 | 0,003 | 4,0 |
| P10 | 0,576 | 34,5 | 0,003 | 4,4 |
| P11 | 0,549 | 46,5 | 0,003 | 4,9 |
| P12 | 0,569 | 6,0 | 0,003 | 3,2 |
| P13 | 0,538 | 6,0 | 0,003 | 3,2 |
| P14 | 0,493 | 4,5 | 0,003 | 3,2 |
| P15 | 0,450 | 3,1 | 0,003 | 3,1 |
| P16 | 0,706 | 27,1 | 0,003 | 4,1 |
| P17 | 0,567 | 16,6 | 0,003 | 3,7 |
| P18 | 0,506 | 2,3 | 0,003 | 2,3 |
| P19 | 0,534 | 2,3 | 0,003 | 2,3 |
| P20 | 0,513 | 82,0 | 0,003 | 6,3 |
| P21 | 0,529 | 53,0 | 0,003 | 5,1 |
| P22 | 0,589 | 39,5 | 0,003 | 4,6 |
| P23 | 0,584 | 12,5 | 0,003 | 3,5 |
| P24 | 0,605 | 11,5 | 0,003 | 3,5 |
| P25 | 0,480 | 1,8 | 0,003 | 1,8 |
| P26 | 0,555 | 2,0 | 0,003 | 2,0 |
| P27 | 0,579 | 24,5 | 0,003 | 4,0 |
| P28 | 0,531 | 9,5 | 0,003 | 3,4 |
| P29 | 0,695 | 33,0 | 0,003 | 4,3 |
| P30 | 0,683 | 27,5 | 0,003 | 4,1 |
| P31 | 0,720 | 27,5 | 0,003 | 4,1 |
| P32 | 0,590 | 20,5 | 0,003 | 3,8 |
| P33 | 0,589 | 16,0 | 0,003 | 3,6 |
| P34 | 0,590 | 11,0 | 0,003 | 3,4 |
| P35 | 0,723 | 21,5 | 0,003 | 3,9 |
| P36 | 0,782 | 27,0 | 0,003 | 4,1 |
| P37 | 0,577 | 70,5 | 0,003 | 5,8 |
| P38 | 0,549 | 83,0 | 0,003 | 6,3 |
| P39 | 0,591 | 7,0 | 0,003 | 3,3 |
| P40 | 0,577 | 11,5 | 0,003 | 3,5 |
| P41 | 0,771 | 45,0 | 0,003 | 4,8 |
| P42 | 0,677 | 33,0 | 0,003 | 4,3 |
| P43 | 0,513 | 12,5 | 0,003 | 3,5 |
| P44 | 0,513 | 12,5 | 0,003 | 3,5 |
| P45 | 0,598 | 41,5 | 0,003 | 4,7 |
| P46 | 0,621 | 7,0 | 0,003 | 3,3 |
| P47 | 0,603 | 45,5 | 0,003 | 4,8 |
| P48 | 0,665 | 13,5 | 0,003 | 3,5 |
| P49 | 0,627 | 27,5 | 0,003 | 4,1 |
| P50 | 0,619 | 34,0 | 0,003 | 4,4 |
| P51 | 0,618 | 59,7 | 0,003 | 5,4 |
| P52 | 0,604 | 260,6 | 0,003 | 13,4 |
| P53 | 0,748 | 225,7 | 0,003 | 12,0 |
| P54 | 0,887 | 112,4 | 0,003 | 7,5 |
| P55 | 0,618 | 126,7 | 0,003 | 8,1 |
| P56 | 0,550 | 30,5 | 0,003 | 4,2 |
| P57 | 0,509 | 7,0 | 0,003 | 3,3 |
| P58 | 0,563 | 18,0 | 0,003 | 3,7 |
| P59 | 0,733 | 57,0 | 0,003 | 5,3 |
| P60 | 0,581 | 39,5 | 0,003 | 4,6 |
| P61 | 0,885 | 32,0 | 0,003 | 4,3 |
| P62 | 0,731 | 132,0 | 0,003 | 8,3 |
| P63 | 0,652 | 27,5 | 0,003 | 4,1 |
| P64 | 0,695 | 25,0 | 0,003 | 4,0 |
| P65 | 0,614 | 19,5 | 0,003 | 3,8 |
| P66 | 0,652 | 3,1 | 0,003 | 3,1 |
| P67 | 0,686 | 4,0 | 0,003 | 3,2 |
| P68 | 0,632 | 27,0 | 0,003 | 4,1 |
| P69 | 0,753 | 32,5 | 0,003 | 4,3 |
| P70 | 0,753 | 32,5 | 0,003 | 4,3 |
| P71 | 0,625 | 57,0 | 0,003 | 5,3 |
| P72 | 0,609 | 4,5 | 0,003 | 3,2 |
| P73 | 0,584 | 4,0 | 0,003 | 3,2 |
| P74 | 0,710 | 4,5 | 0,003 | 3,2 |
| P75 | 0,629 | 3,5 | 0,003 | 3,1 |
| P76 | 0,624 | 3,5 | 0,003 | 3,1 |
| P77 | 0,642 | 53,0 | 0,003 | 5,1 |
| P78 | 0,642 | 53,0 | 0,003 | 5,1 |
| P79 | 0,739 | 20,5 | 0,003 | 3,8 |
| P80 | 0,767 | 32,5 | 0,003 | 4,3 |
| P81 | 0,757 | 26,5 | 0,003 | 4,1 |
| P82 | 0,568 | 9,5 | 0,003 | 3,4 |
| P83 | 0,551 | 6,5 | 0,003 | 3,3 |
| P84 | 0,716 | 10,5 | 0,003 | 3,4 |
| P85 | 0,854 | 43,5 | 0,003 | 4,7 |
| P86 | 0,792 | 57,5 | 0,003 | 5,3 |
| P87 | 0,724 | 57,5 | 0,003 | 5,3 |
| P88 | 1,089 | 32,8 | 0,003 | 4,3 |
| P89 | 0,576 | 2,3 | 0,003 | 2,3 |
| P90 | 0,601 | 2,3 | 0,003 | 2,3 |
| P91 | 0,578 | 7,0 | 0,003 | 3,3 |
| P92 | 0,620 | 27,7 | 0,003 | 4,1 |
| P93 | 0,681 | 7,6 | 0,003 | 3,3 |
| P94 | 0,720 | 50,1 | 0,003 | 5,0 |
| P95 | 0,504 | 2,0 | 0,003 | 2,0 |
| P96 | 0,762 | 11,6 | 0,003 | 3,5 |
| P97 | 0,608 | 8,5 | 0,003 | 3,3 |
| P98 | 0,741 | 57,6 | 0,003 | 5,3 |
| P99 | 0,710 | 33,0 | 0,003 | 4,3 |
| P100 | 0,515 | 4,5 | 0,003 | 3,2 |
| P101 | 0,533 | 14,5 | 0,003 | 3,6 |
| P102 | 0,904 | 45,3 | 0,003 | 4,8 |
| P103 | 0,546 | 21,0 | 0,003 | 3,8 |
| P104 | 0,788 | 49,0 | 0,003 | 5,0 |
| P105 | 0,523 | 4,0 | 0,003 | 3,2 |
| P106 | 0,601 | 18,5 | 0,003 | 3,7 |
| P107 | 0,544 | 7,0 | 0,003 | 3,3 |
| P108 | 0,450 | 20,5 | 0,003 | 3,8 |
| P109 | 0,530 | 38,5 | 0,003 | 4,5 |
| P110 | 0,646 | 13,5 | 0,003 | 3,5 |
| P111 | 0,719 | 18,0 | 0,003 | 3,7 |
| P112 | 0,652 | 6,5 | 0,003 | 3,3 |
| P113 | 0,743 | 133,5 | 0,003 | 8,3 |
| P114 | 0,785 | 158,5 | 0,003 | 9,3 |
| P115 | 1,035 | 158,0 | 0,003 | 9,3 |
| P116 | 0,798 | 13,5 | 0,003 | 3,5 |
| P117 | 0,873 | 33,0 | 0,003 | 4,3 |
| P118 | 0,698 | 131,0 | 0,003 | 8,2 |
| P119 | 0,731 | 159,0 | 0,003 | 9,4 |
| P120 | 0,610 | 37,5 | 0,003 | 4,5 |
| P121 | 0,630 | 18,5 | 0,003 | 3,7 |
| P122 | 0,901 | 30,5 | 0,003 | 4,2 |
| P123 | 0,745 | 6,5 | 0,003 | 3,3 |
| P124 | 0,448 | 11,0 | 0,003 | 3,4 |
| P125 | 0,797 | 8,0 | 0,003 | 3,3 |
| P126 | 0,903 | 7,5 | 0,003 | 3,3 |
| P127 | 0,665 | 7,5 | 0,003 | 3,3 |
| P128 | 0,573 | 46,0 | 0,003 | 4,8 |
| P129 | 0,999 | 28,0 | 0,003 | 4,1 |
| P130 | 0,716 | 157,8 | 0,003 | 9,3 |
| P131 | 0,644 | 83,6 | 0,003 | 6,3 |
| P132 | 0,687 | 34,0 | 0,003 | 4,4 |
| P133 | 0,764 | 21,0 | 0,003 | 3,8 |
| P134 | 0,664 | 34,0 | 0,003 | 4,4 |
| P135 | 0,761 | 6,0 | 0,003 | 3,2 |
| P136 | 0,760 | 2,5 | 0,003 | 2,5 |
| P137 | 0,533 | 47,5 | 0,003 | 4,9 |
| P138 | 0,827 | 24,1 | 0,003 | 4,0 |
| P139 | 0,491 | 5,5 | 0,003 | 3,2 |
| P140 | 0,727 | 57,0 | 0,003 | 5,3 |
| P141 | 0,593 | 8,5 | 0,003 | 3,3 |
| P142 | 0,566 | 13,0 | 0,003 | 3,5 |
| P143 | 0,576 | 2,5 | 0,003 | 2,5 |
| P144 | 0,653 | 10,5 | 0,003 | 3,4 |
| P145 | 0,612 | 14,0 | 0,003 | 3,6 |
| P146 | 0,550 | 11,0 | 0,003 | 3,4 |
| P147 | 0,603 | 22,5 | 0,003 | 3,9 |
| P148 | 0,710 | 8,0 | 0,003 | 3,3 |
| P149 | 0,756 | 104,0 | 0,003 | 7,2 |
| P150 | 0,747 | 56,5 | 0,003 | 5,3 |
| P151 | 0,944 | 56,5 | 0,003 | 5,3 |
| P152 | 0,637 | 29,5 | 0,003 | 4,2 |
| P153 | 0,533 | 4,5 | 0,003 | 3,2 |
| P154 | 0,575 | 23,0 | 0,003 | 3,9 |
| P155 | 0,555 | 45,5 | 0,003 | 4,8 |
| P156 | 0,579 | 4,5 | 0,003 | 3,2 |
| P157 | 0,584 | 7,0 | 0,003 | 3,3 |
| P158 | 0,633 | 63,5 | 0,003 | 5,5 |
